# The Specificity of the *FOXL2* c.402C**>**G Somatic Mutation: A Survey of Solid Tumors

**DOI:** 10.1371/journal.pone.0007988

**Published:** 2009-11-24

**Authors:** Kasmintan A. Schrader, Bella Gorbatcheva, Janine Senz, Alireza Heravi-Moussavi, Nataliya Melnyk, Clara Salamanca, Sarah Maines-Bandiera, Susanna L. Cooke, Peter Leung, James D. Brenton, C. Blake Gilks, John Monahan, David G. Huntsman

**Affiliations:** 1 Department of Pathology and Laboratory Medicine, University of British Columbia, Vancouver, British Columbia, Canada; 2 Centre for Translational and Applied Genomics, British Columbia Cancer Agency, Vancouver, British Columbia, Canada; 3 Novartis Institute for Biomedical Research, Cambridge, Massachusetts, United States of America; 4 Cancer Research, Cambridge Research Institute, Cambridge, United Kingdom; 5 Department of Obstetrics and Gynecology, University of British Columbia, Child and Family Research Institute, Vancouver, British Columbia, Canada; 6 Genetic Pathology and Evaluation Centre, Vancouver General Hospital, Vancouver, British Columbia, Canada; Innsbruck Medical University, Austria

## Abstract

**Background:**

A somatic mutation in the *FOXL2* gene is reported to be present in almost all (97%; 86/89) morphologically defined, adult-type, granulosa-cell tumors (A-GCTs). This *FOXL2* c.402C>G mutation changes a highly conserved cysteine residue to a tryptophan (p.C134W). It was also found in a minority of other ovarian malignant stromal tumors, but not in benign ovarian stromal tumors or unrelated ovarian tumors or breast cancers.

**Methodology/Principal Findings:**

Herein we studied other cancers and cell lines for the presence of this mutation. We screened DNA from 752 tumors of epithelial and mesenchymal origin and 28 ovarian cancer cell lines and 52 other cancer cell lines of varied origin. We found the *FOXL2* c.402C>G mutation in an unreported A-GCT case and the A-GCT-derived cell line KGN. All other tumors and cell lines analyzed were mutation negative.

**Conclusions/Significance:**

In addition to proving that the KGN cell line is a useful model to study A-GCTs, these data show that the c.402C>G mutation in *FOXL2* is not commonly found in a wide variety of other cancers and therefore it is likely pathognomonic for A-GCTs and closely related tumors.

## Introduction

Malignant adult ovarian granulosa-cell tumors (A-GCTs) are malignant sex cord-stromal tumors known for their genomic stability and varied prognosis [1]. Until recently, there has been little insight into the molecular characteristics of A-GCTs. Using whole-transcriptome paired-end RNA sequencing, we identified a somatic missense mutation (c.402C>G, p. Cys134Trp) in the Forkhead transcription factor gene, *FOXL2* [2]. This mutation was present in 97% of 89 morphologically identified A-GCTs [2]. Foxl2 has been shown to be crucial for granulosa-cell differentiation [3]. This was the first association of a somatic mutation in *FOXL2* associated with cancer, however aberrant expression of Foxl2 has been reported in juvenile granulosa-cell tumor of the testis [4]. The mutation was also found at a lower frequency in two other related ovarian stromal tumors; 21% (3/14) thecomas and 10% (1/10) juvenile-type GCTs were mutation positive [2]. This single, recurrent mutation suggests that it is characteristic of granulosa-cell tumors, and its high frequency implies that it is potentially a driver in disease initiation.

To determine the specificity of this somatic mutation, high resolution melting or polymerase chain reaction (PCR) -based allelic discrimination was used to screen a diverse collection of tumors and ovarian tumor cell lines. Additional cytogenetic analysis was performed to demonstrate the stable karyotype of the A-GCT cell line, KGN [5].

## Materials and Methods

Samples for the high resolution melt assay were purchased as either DNA or tissue blocks from vendors who provided unlinked anonymized specimens collected in accordance with applicable review boards approval, regulations and laws. Novartis does not require an ethical review committee for samples collected in this manner. Control DNA, used to validate the high resolution melt assay, was extracted from anonymized tumor specimens compiled by the frozen tumor bank, OvCaRe (Ovarian Cancer Research), under written informed consent. Approval for analysis of these samples for the *FOXL2* mutation was obtained through the British Columbia Cancer Agency's research ethics board.

Seven hundred and fifty-two tumor DNA samples, of epithelial and mesenchymal origin (Table 1) were screened with a high resolution melting assay run on the LightScanner™ instrument (Idaho Technology Inc., Salt Lake City, Utah) [6], [7]. For each tumor block, malignant cells composed >50% of the cellularity and matched normal adjacent tissue was available for all cases. The assay was designed to detect sequence variants in the region from Ile102 to Phe138 in Foxl2 (NP_075555.1). Since *FOXL2* is a single exon gene, PCR primers were placed in the coding region (forward primer 5′ AGAAGGGCTGGCAAAATAGC, reverse primer 5′ GCCGGTAGTTGCCCTTCT) resulting in a 150 base pair amplicon.

**Table 1:** Summary of tumor types screened by High Resolution Melt Curve Analysis (HRM).

	Total cases n = 752 (excluding controls)	Normal by HRM	Confirmed positive for *FOXL2* c.402C>G mutation out of HRM positive cases
Ovarian cancer negative controls	14	11	0/3*
Ovarian A-GCT positive controls (including an unreported A-GCT case and the A-GCT cell line, KGN)	13	0	13/13
Bladder Cancer	40	40	
Breast Cancer	74	71	0/3*
Carcinoid Cancer	8	8	
Cervical Cancer	16	16	
Colorectal Cancer	77	75	0/2*
Endometrial Cancer	12	12	
Esophageal Cancer	21	21	
Gastric Cancer	90	89	0/1*
Head & Neck Cancer	28	26	0/2*
Hepatic (HCC & Cholangiocarcinoma)	14	14	
Lung Cancer (All types)	125	123	0/2*
Melanoma	31	31	
Ovarian Cancer	32	32	
Pancreatic Cancer	4	4	
Prostate Cancer	37	37	
Renal Cancer	52	51	0/1*
Leiomyosarcoma	15	15	
Malignant fibrous histiocytoma-pleomorphic sarcoma	8	8	
Rhabdomyosarcoma	2	2	
Liposarcoma	4	4	
Fibrosarcoma	1	1	
Testicular Cancer	19	18	0/1*
Thyroid Cancer	42	42	

Sequence data is available for all screen positive samples.

* Variants seen on HRM screen but not confirmed by sequencing (HRM false positive results).

The primary screen used whole genome amplified (Qiagen Repli-G kit) DNA derived from frozen tissue blocks of untreated primary tumors. All samples which had an aberrant melting curve or which failed to amplify in the initial screen were followed up with a repeat HRM assay using unamplified DNA prepared from tumor and adjacent normal tissue. Tumor samples which were repeat positive for an aberrant melting curve were sequenced in duplicate, and the resulting sequence trace files were analyzed for mutations using the phrap/phred/consed software package (www.phrap.org). DNA from 27 ovarian tumor samples previously genotyped for the mutation using a previously validated TaqMan real-time PCR-based allelic discrimination assay (Applied Biosystems, Foster City, CA) specific for the *FOXL2* c.402C>G mutation [2] were used to validate the performance of the HRM assay. This included an unreported A-GCT case and the cell line KGN.

To establish the specificity of the *FOXL2* c.402C>G mutation in ovarian cancer cell lines, we used the same TaqMan real-time PCR-based allelic discrimination assay to genotype 28 ovarian cancer cell lines and 52 cancer cell lines of different tissue origin for the *FOXL2* c.402C>G mutation (Table S1).

To assess the cytogenetic profile of KGN, we utilized 24-color fluorescence *in situ* hybridization (FISH) (24XCyte, MetaSystems, Cat. D-0125-120-MC) and analyzed the results using the Axioplan 2, Zeiss,(MetaSystems, Isis), camera VAC-30054.

## Results

All 11 previously reported *FOXL2* c.402C>G mutation-positive A-GCT specimens as well as an unreported A-GCT case and the A-GCT cell line, KGN, validated the HRM assay by demonstrating a variant melt curve distinct from the common (wild-type) pattern. None of the 14 *FOXL2* c.402C>G mutation negative samples exhibited this variant melt profile. However, three of the 10 high grade serous ovarian cancers showed an alternative variant profile; sequencing confirmed them to be false positives.

The primary screen of 752 whole genome amplified tumor DNA samples yielded 24 samples (∼4%) with a variant profile, distinct from that seen in association with the *FOXL2* c.402C>G mutation-positive A-GCT specimens, as well as 41 with an indeterminate profile and 29 samples that failed. The secondary screen was performed on this set of 94 samples using unamplified genomic DNA derived from tumors and matching normal specimens. Eighty-two of the samples were found to be false positives where there was no variant profile seen between the tumor and normal DNA. Twelve of the samples remained indeterminate and were subsequently sequenced and confirmed to be false positives.

**Figure 1:**
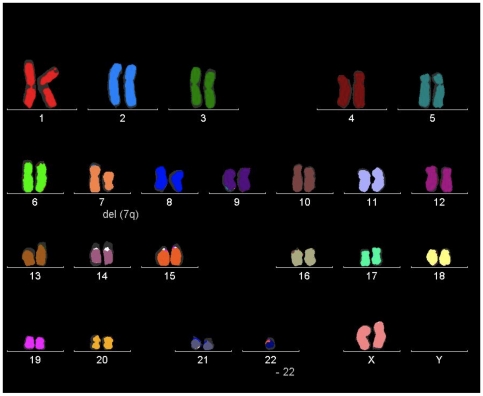
Cytogenetic analysis of the KGN cell line. 24-color fluorescence *in situ* hybridization (FISH) demonstrates the tumor cell line's stable karyotype 45, XX, 7q-, -22 consistent with the original publication [5]. *FOXL2* is located at 3q23. Images were obtained using the Axioplan 2, Zeiss, (MetaSystems, Isis), camera VAC-30054.

The granulosa-cell line KGN, which was derived through long-term passage of a recurrent A-GCT [5], was the only cell line found to harbor the mutation. The mutation was not present in an SVOG granulosa-cell line, immortalized by SV40 [8] or 26 other ovarian cancer derived cell lines. Unlike most ovarian cancer derived cell lines KGN shows relative genomic stability (Figure 1). In addition to deletion of 7q, it is monosomic for chromosome 22 which is the most frequent cytogenetic abnormality seen in A-GCTs [9]; another feature demonstrating its similarities to A-GCTs.

## Discussion

Loss-of-function germline mutations in *FOXL2* are associated with blepharophimosis–ptosis–epicanthus–inversus syndrome [BPES;OMIM#110100]; an autosomal dominant developmental disorder characterized by eyelid malformations and premature ovarian failure due to a dysfunction of granulosa-cells [10]. The *FOXL2* c.402C>G mutation is seen in the heterozygous state in most A-GCTs. Unlike in BPES, where germline *FOXL2* mutations are spread across the gene [11], the somatic *FOXL2* mutation in A-GCTs involves the same base pair in all cases. This favors a specific functional consequence such as a dominant negative effect or a change or gain of function as opposed to a generic loss of function and the ultimate impact of this mutation is oncogenic. Additionally, immunohistochemical data indicating that Foxl2 expression is maintained in the nuclei in A-GCTs, that were heterozygous or appeared to be hemizygous or homozygous for the mutation, implies that this mutation does not affect protein localization [2].

Analysis of 28 various ovarian cancer-derived cell lines demonstrated that the mutation was only present in the granulosa-cell tumor cell line, KGN, suggesting that it is molecularly akin to A-GCTs. The presence of the missense mutation in the well-characterized A-GCT cell line, KGN, is in keeping with the high frequency of the somatic mutation in A-GCTs and supports the use of this cell model to study the properties of this ovarian sex cord stromal tumor. This cell line has been used in a number of elegant studies which have addressed the question of the function of Foxl2 [12] and the effects of *FOXL2* missense, haploinsufficient or hypomorphic mutations associated with BPES [13]–[15]. Further dissection of these phenomena with attention to the possible confounding effects of this mutation in one copy of the endogenous gene may elucidate the function of this missense mutation in the granulosa-cell tumor.

The absence of the *FOXL2* c.402C>G mutation in this large series of common epithelial malignancies such as lung, colorectal, breast, gastric, bladder, thyroid, prostate, melanoma and ovarian carcinoma, in addition to a range of less frequent tumors, implies a high specificity of this recurrent mutation for ovarian sex cord stromal tumors. This study does not exclude the possibility that the mutation could be found in other rare or related neoplasms such as testicular stromal tumors. As the mutation was not found in non-GCT ovarian tumor cell lines and the SV40 transformed granulosa-cell line, SVOG, provides further support of its likely role in A-GCT disease initiation. Considering the extremely high frequency of this mutation in morphologically selected A-GCTs (97%) [2], these data provide further evidence suggesting that the mutation is also specific for this tumor type and could be useful as a diagnostic test. Further studies will be required to determine the relevance of the mutation in other sex cord stromal tumors of the ovary, however, it is possible that all mutation positive tumors could ultimately be considered to be a single entity of which the major component would be A-GCTs.

## Supporting Information

Table S1 Cell lines screened by TaqMan real-time PCR-based allelic discrimination assay for the *FOXL2* c. 402 C>G mutation. Ovarian cancer cell lines are italicized and ovarian granulosa-cell-derived lines are underlined.
(0.02 MB XLS)
